# Genetic Diversity and Genome-Wide Association Study of Total Phenolics, Flavonoids, and Antioxidant Properties in Potatoes (*Solanum tuberosum* L.)

**DOI:** 10.3390/ijms252312795

**Published:** 2024-11-28

**Authors:** Haroon Rasheed, Bowen Deng, Daraz Ahmad, Jinsong Bao

**Affiliations:** Institute of Nuclear Agricultural Sciences, College of Agriculture and Biotechnology, Zhejiang University, Hangzhou 310058, China; haroonzju@zju.edu.cn (H.R.); bwdeng@zju.edu.cn (B.D.); darazahmad786@gmail.com (D.A.)

**Keywords:** potato, genetic diversity, antioxidant properties, nutritional quality, environment, genome-wide association study

## Abstract

Genetic diversity of nutritional quality traits is crucial for potato breeding efforts to develop better varieties for the diverse market demands. In this study, the genetic diversity of 104 potato genotypes was estimated based on nutritional quality traits such as color parameters, total phenolic content, total flavonoid content, 2,2-Diphenyl-1-picrylhydrazyl (DPPH), and 2,2-azino-bis-(3-ethylbezothiazoline-6-sulphonic acid) radical scavenging potential across two environments. The results indicated that environment II, Hangzhou 2020, exhibited higher bioactive compounds and antioxidant properties than environment I, Hangzhou 2019. The colored potato accessions exhibited higher levels of total phenolic content, total flavonoid content, DPPH, and ABTS activities than the white potato accessions, indicating the superiority of the colored to white potato accessions. The genome sequencing identified 1,101,368 high-quality single-nucleotide polymorphisms (SNPs), and 141,656 insertion/deletions (Indels). A population structure analysis revealed that genotypes can be divided into two subpopulations. Genome-wide association studies (GWAS) identified 128 significant SNPs associated with potato’s color, total phenolic content, total flavonoid content, and antioxidant properties. Thus, the study provides new opportunities for strategic breeding and marker-assisted selection of ideal varieties and favorable alleles to enhance bioactive compounds and health-beneficial properties.

## 1. Introduction

Potato (*Solanum tuberosum* L.) belongs to the *Solanaceae* family, comprising around 4000 well-known varieties, including 10 cultivated and 200 wild species worldwide [1,2]. Potatoes contribute significantly to human health by providing various health-beneficial compounds [3,4], such as phenolic acids and flavonoids, thereby enhancing their nutritional values [5,6].

Potatoes exhibit a wide genetic diversity, indicating significant variability in nutritional composition [1,7]. Among the diverse range of health-beneficial compounds, phenolic acids are extensively identified in potatoes [8]. Chlorogenic acid is the most frequent, constituting approximately 90% of the total phenolic acids, although caffeic, coumaric, protocatechuic, vanillic, ferulic, syringic, sinapic, and gallic acids have also been identified in potato varieties [2,9,10,11]. The total phenolic acids in potatoes range from 54 to 359 mg gallic acid equivalent (GAE)/100 g DW in white potatoes, from 152 to 261 mg GAE/100 g DW in red potatoes, and in purple potatoes, they range from 162 to 510 mg GAE/100 g DW [12]. Flavonoids are also known as the most diverse group of potato polyphenols. They are usually attached to sugars in the form of glycosides, although they can exist as aglycons or free molecules and enhance the color and flavor of fruits and vegetables [13]. The total flavonoid content in various potatoes ranged from 0.06 to 2.29 mg of catechin equivalent per gram of dry weight (mg CE/g DW) [14,15]. The most abundantly found flavonoid in potatoes is catechin, ranging from 43.0 to 204.0 mg/100 g DW [11,16,17,18]. Potato varieties with red and purple colors are rich sources of phenolic compounds compared with white potato varieties [9,19]. The purple-colored potato cultivars have more phenolic and flavonoid content and health-beneficial properties than red potato verities [5,20]. Light-colored potatoes contain flavanols, while colored potatoes contain anthocyanin, which is the most common type of flavonoid [20]. These pigments give potatoes their colors, ranging from red and orange to purple and blue. This is why pigmented potatoes are essential for extracting natural colorants and antioxidants in nutraceutical, pharmaceutical, and other industries [20,21,22].

The health-beneficial properties of potato phenolic compounds and other nutrients have captured the keen interest of nutritionists worldwide [23,24]. The bioactive compounds present in potatoes such as phenolic acids, flavonoids, and other nutrients exhibit antioxidant, anticancer, antidiabetic, anti-obesity, and anti-inflammatory properties, and have the potential to reduce chronic diseases like heart diseases, diabetes, and certain types of cancer [5,6,25,26]. It is well known that the study of natural antioxidants has been popular for many years because they protect against free radicals, thereby preventing various chronic diseases [27,28,29]. The antioxidant properties of potatoes help to reduce DNA damage and inflammation in men [13,30]. Colored-fleshed potatoes are nutritionally more stable as their antioxidant potential is two to three times higher than white-fleshed potatoes [31,32]. Consuming colored potatoes is crucial for age-related diseases, diabetes, and cancer prevention [25,32]. This nutritional profile highlights the significant contribution of potatoes in maintaining good health.

Phenolic compounds are significantly affected by the diverse environmental conditions during tuber development processes [9,33]. A recent study revealed that warmer growing conditions, in which temperature ranges from 19.7 to 34.2 °C and relative humidity ranges from 35.5 to 87.5%, likely facilitate the production of bioactive compounds and enhance biological properties [34]. The production system, location [35], and genotype-by-environment (G × E) interaction in potatoes affect quality traits, especially phenolic acid, flavonoids, and antioxidant properties [34,36,37]. Still, the genetic makeup has more effect on the potato’s bioactive components and antioxidant capacity [21,38]. To choose the most favorable germplasm effectively and quickly, it is necessary to understand the genetic basis of the traits of agronomic importance [39]. Genome-wide association study (GWAS) is an effective and powerful tool to exactly map the complex traits that contribute to the productivity of crops with high resolution [40]. Numerous GWAS approaches have been applied to potato yield and quality improvement. A GWAS analysis was conducted on 237 potato genotypes grown in two environments, focusing on various morphological and agronomic traits, such as potato flower and tuber quality. Using the mixed linear model, 36 genomic regions were identified for the agronomic and morphological traits [41]. The resequencing of 108 potato cultivars revealed 27 million SNPs and 3 million Indels, and the GWAS detected significant genes associated with flowering time, disease resistance, and temperature sensitivity [42]. Various candidate genes for various agronomic traits, including tuber size, small-size tuber weight, and thickness, were further confirmed by transcriptome analysis [42,43]. A GWAS approach also identified 4488 SNP markers significantly associated with total phenolic and ascorbic acid content and the antioxidant properties of 404 potato varieties [37]. The results showed 58 single-locus SNP-trait associations and 28 multi-locus SNP-trait associations for the traits, 8 of which were related to total phenolic and ascorbic acid content and antioxidant properties, along with 7 candidate genes, 4 of which were pleiotropic [37]. Another GWAS analysis identified 18 SNPs strongly linked with chlorogenic acid levels in 271 potato varieties grown in different environments. The study revealed that chlorogenic acid has a major effect on flavor, appearance, and nutrient content in potatoes [44]. The previous studies provide strong evidence regarding the genetic basis of various agronomic, quality, and yield-related traits. However, to the best of our knowledge, there are not enough available data from the literature on the genetic basis of total phenolic, flavonoid, and antioxidant properties in potatoes. Thus, the aims and objectives of the present study are (1) to identify genetic variations with key nutritional traits in potatoes, such as phenolic content, flavonoid content, and antioxidant properties in potato accessions; (2) to understand population structure and linkage disequilibrium of potato accessions; and (3) to identify genetic markers and candidate genes associated with the variations in these nutritional quality traits. The results of the current study may further contribute to our understanding of the genetic diversity and nutritional composition of potatoes.

## 2. Results

### 2.1. Genotyping by Sequencing Identified High-Quality SNPs and Indels Across the Genome

**Figure 1:**
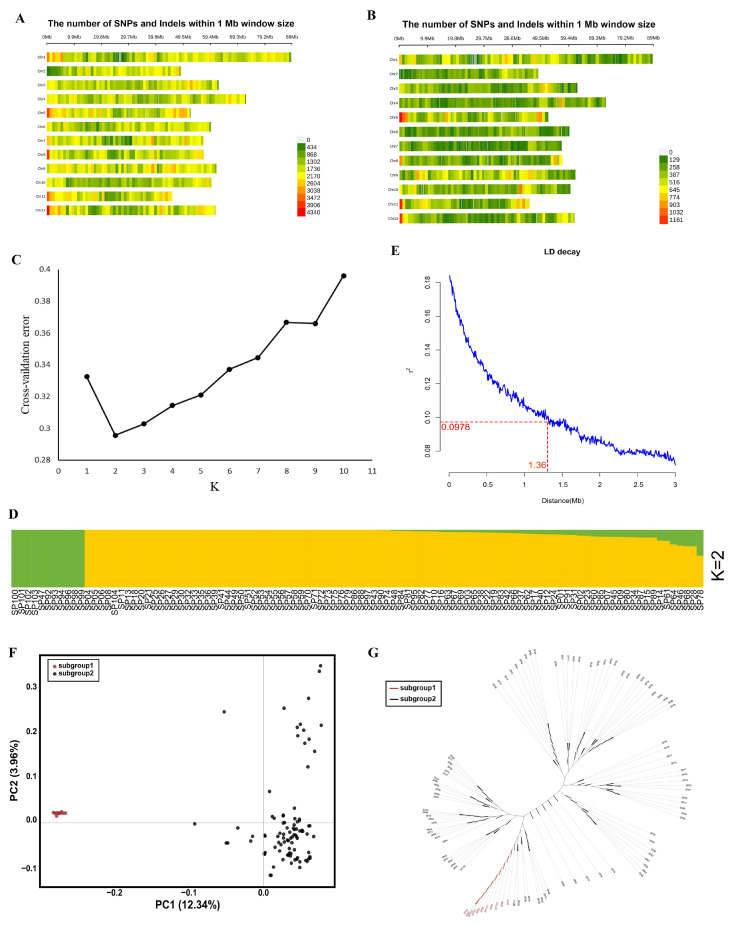
Genetic diversity and population structure of 104 potato accessions. (**A**,**B**) SNP distribution and density in potato’s chromosomes. (**C**) ADMIXTURE analysis of cross-validation, K = 1 – 10. (**D**) Population structure based on K = 2 for the whole panel of potato accessions. (**E**) Linkage disequilibrium plot for the tested genotypes. (**F**) Principal component analysis (PCA) denotes the first two principal components of the population, subpopulation 1 (red dots) and subpopulation 2 (black dots). (**G**) Neighbor-joining tree constructed using a P-distance matrix for 104 accessions.

The diverse panel of 104 potato accessions was subjected to double-digested Restriction-site Associated DNA sequencing (ddRAD-seq). These results were then aligned with the potato genome (PGSC-DM_v4.03) using the BWA-MEM. After SNP calling, 1,101,368 high-quality SNPs with minor allele frequency higher than 0.05 and missing rate less than 0.15 were generated, distributed as follows: 75,533 exonic regions, 128,133 intronic regions, 38,131 in untranslated regions (UTRs), and 682,604 in the intergenic regions (Table 1). Among these SNPs, 39,547 were non-synonymous, 34,814 were synonymous, 216 affected splice sites, 1064 led to stop-gain mutations, and 125 resulted in stop-loss mutations within the coding regions. Similarly, 141,656 high-quality insertion/deletions (Indels) were identified (Table 1), including 3359 exonic, 21,696 intronic, 8703 untranslated regions (UTRs), and 76,580 intergenic indels. These included 92 splicing, 80 stop-gain, and 9 stop-loss Indels within the coding areas. The number of SNPs analyzed in the panel varied across the 12 potato chromosomes, with chromosome 1 having the highest number (134,350) and chromosome 2 having the lowest number (69,709) (Figure 1A). After adjusting heterozygosity in SNP and Indel markers (<0.2), the remaining 226,487 SNP and 22,115 Indel sites were merged to construct the density plot (Figure 1B).

### 2.2. Genetic Diversity, Population Structure, and Linkage Disequilibrium of Potatoes

Cross-validation error analysis revealed that the error peak was the lowest at K = 2, indicating that the grouping was optimal. This indicated that potato accessions could be divided into two subpopulations (Figure 1C), where one subpopulation represents 11 potato genotypes while the second subpopulation contains 93 (Figure 1D). The linkage disequilibrium (LD) plot indicated LD attenuation distances of all the tested genotypes were 1.36 Mbs (Figure 1E). Principal component analysis (PCA) described 16.3% of the genetic variations in the first two principal components (Figure 1F). However, from the unrooted tree constructed using the unweighted neighbor-joining method, it was indicated that all the potato genotypes were clustered into different groups (Figure 1G).

### 2.3. Diversity of Color Parameters

The color parameters of potato accessions, including L*, a*, b*, C*, and H°, were analyzed (Table 2). The total mean lightness (L*) values for fresh potatoes were 66.27 and 67.92, while for potato powder, they were 87.03 and 88.87, indicating that fresh potatoes have darker color than potato fine powder. Redness (a*) values were slightly lower in fresh potatoes than in potato fine powder. Yellowness (b*) values and color intensity (C*) values were significantly higher in fresh potatoes than in potato fine powder, while the hue angle (H°) values were consistent in colored and white potatoes across the two environments. Our results signify a wide genetic diversity among the tested potato accessions.

### 2.4. Total Phenolic and Flavonoid Content in Potatoes

**Figure 2:**
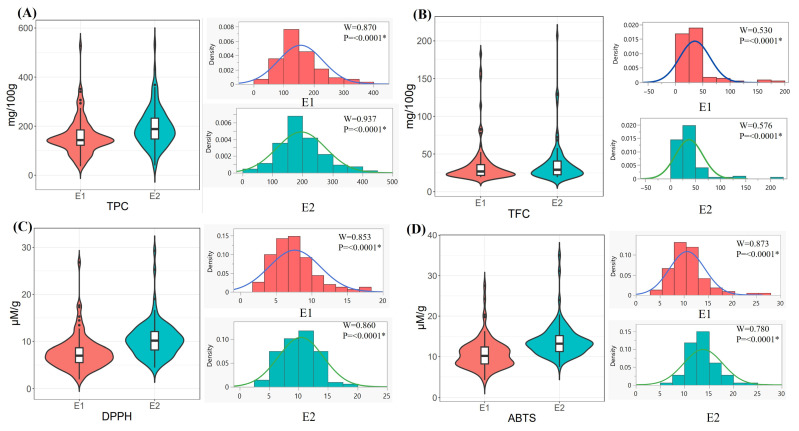
Mean distributions of total phenolic content, total flavonoid content, DPPH, and ABTS activities of potatoes. (**A**) TPC: total phenolic content expressed as mg GAE/100 g; (**B**) TFC: total flavonoid content expressed as mg/g CE/100 g; (**C**) DPPH: 2,2-Diphenyl-1-picrylhydrazyl; and (**D**) ABTS: 2,2-azino-bis-(3-ethylbenzothiazoline-6-sulphonic acid) expressed as µM TE/g of potato powder; W = Shapiro–Wilk test statistics; * *p* < 0.05.

A wide-ranging total phenolic and flavonoid content was assessed in potatoes in two consecutive environments (Figure 2A,B). The test statistics (W) as per the Shapiro–Wilk test indicated that most of the total phenolic content results were in the range of 0.8 to 0.9, indicating normal or near-normal distribution. The total phenolic content values were higher in colored potato samples than in non-colored potatoes, as previously reported [45,46]. Phenolic content in colored potatoes ranged from 306.9 to 531.78 mg GAE/100 g of potato powder. White potato samples had 36.9 to 368.4 mg GAE/100 g of potato powder. Our findings showed that environmental interactions affected phenolic content over the two seasons, ranging from 36.9 to 527.4 mg GAE/100 g of potato powder in environment I, and 40.51 to 531.78 in environment II (Table 3). The statistical results for total flavonoid content in environment I (0.53) and environment II (0.57) indicated significant evidence against normality. Colored potatoes had a wide range of flavonoid content, from 22.93 to 182.46 mg CE/100 g of potato powder, whereas white potatoes had a lower range, ranging from 19.22 to 160.32 mg CE/100 g in environment I. The variations were still observed in environment II, with colored potatoes exhibiting flavonoid content ranging from 130.02 to 206.90 mg CE/100 g and white potatoes revealed a range of 19.29 to 128.79 mg CE/100 g for potato powder (Table 3). Thus, the overall findings revealed some environmental effects, but colored potatoes had much higher total phenolic and flavonoid contents than white potatoes, indicating the superiority of colored potatoes over white potatoes in both tested environments.

### 2.5. Antioxidant Properties of Potato Population

The antioxidant properties measured for DPPH and ABTS in colored potato samples were significantly higher than those in non-colored potato samples in both environments (Table 3). The test statistics (W) for DPPH were around 0.86 and for ABTS, around 87, indicating some consistency with normal distribution (Figure 2C,D). The average results indicated that DPPH activity ranged from 1.95 to 26.82 µM TE/g in environment I and from 4.46 to 29.3 µM TE/g of potato powder in environment II, indicating a broader range of DPPH radical scavenging activities across the two seasons. ABTS activity ranged from 4.23 to 27.99 µM TE/g in environment I and 7.47 to 28.03 µM TE/g of potato powder in environment II. These findings highlight the significant variations in DPPH and ABTS radical scavenging activities across different environments, indicating the potential impact of color and environmental factors on potato accessions.

### 2.6. Correlation Analysis of Potato Population

**Figure 3:**
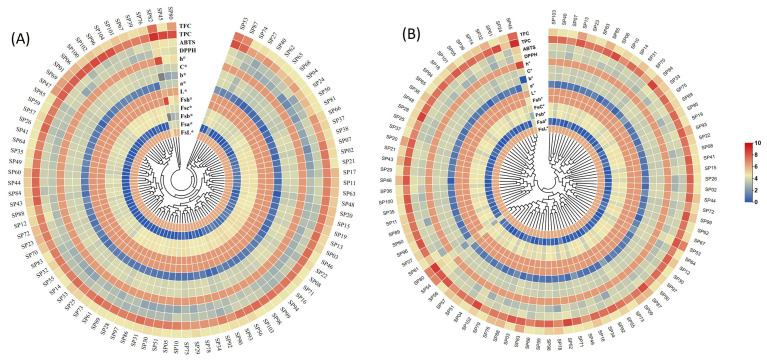
Circular heat map based on Pearson’s correlation of 104 potato accessions in two environments. (**A**) environment I and (**B**) environment II. TFC, total flavonoid content; TPC, total phenolic content (mg/100 g); DPPH, 2,2-Diphenyl-1-picrylhydrazyl; and ABTS, 2,2-azino-bis-(3-ethylbenzothiazoline-6-sulphonic acid) (µM TE/g of potato powder). Color ranges from dark blue to dark red indicate lower to higher levels of color parameters, TPC, TFC, DPPH, and ABTS.

Pearson correlation revealed that the highest significant positive correlation was observed between DPPH, ABTS, total phenolic content, and total flavonoid content for all the tested samples in both environments (Table 4). Across the two seasons, L* and b* were significantly negatively correlated with DPPH, ABTS, total phenolic content, and total flavonoid content, while a* and H° were significantly positively correlated with DPPH, ABTS, total phenolic, and total flavonoid content. The values of C* were mostly non-significant and negatively correlated throughout the panel. Moreover, hierarchical cluster analysis based on person correlation also represents widespread nutritional information of the diverse panel of potatoes across the two environments (Figure 3). Clustering data revealed that colored potato accessions were the highest among all the tested potatoes in both environments (Figure 3A,B). The overall correlation results indicated that the environment has some effects, while the diverse range of colors plays a pivotal and significant role in the nutritional composition and health-beneficial properties of potatoes.

### 2.7. Analysis of Variance and Broad-Sense Heritability of Color, DPPH, ABTS, TPC, and TFC in Potato Population

The mean square value represents potato nutritional quality traits such as the color parameter (L*, a*, b*, C*, H°) of fine powder and fresh potatoes (Table 2), total phenolic content, total flavonoid content, and their antioxidant properties (DPPH and ABTS radical scavenging properties) of two consecutive seasons (Table 3). The mean square values showed the highest significant values at *p* < 0.01 at the genotype level and genotype–environment interaction for all the tested traits. Therefore, it can be concluded that genotypic and environment–genotype interactions can influence the nutritional qualities of potatoes. However, a few traits, including fresh potato color a*, b* C*, and H° and potato powder color a*, were significantly influenced by genetic factors compared to environmental factors as per potatoes’ broad-sense heritability (H^2^) values.

### 2.8. Genome-Wide Association Studies (GWAS) of Diverse Potato Population

**Figure 4:**
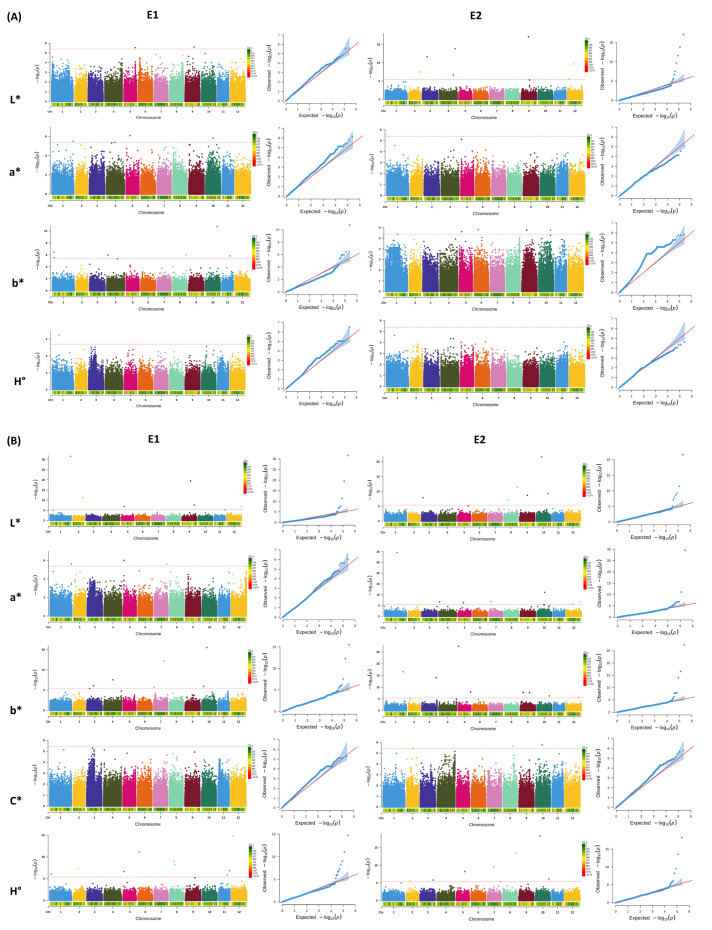
Manhattan plots represent a genome-wide association study regarding color parameters in 104 potato accessions across two growing seasons. E1 represents environment I and E2 represents environment II. (**A**) Color parameters on a fresh basis of potatoes and (**B**) color parameters of potato fine powder (L* = lightness, a* = redness, b* = yellowness, C* = chroma, and H° = hue angle). The x-axis represents the chromosome number and the position of each SNP, while the y-axis represents the negative logarithm (−log10) of the *p*-values for each SNP. The broken lines represent significant thresholds 4.02 × 10^−6^. The heat maps at the bottom of each chromosome represent SNP density. The QQ plot on the left shows the observed vs. expected −log10 values. The diagonal line shows the predicted distribution whereas the other line indicates the observed p-values. The deviation from the diagonal line indicates a significant association.

**Figure 5:**
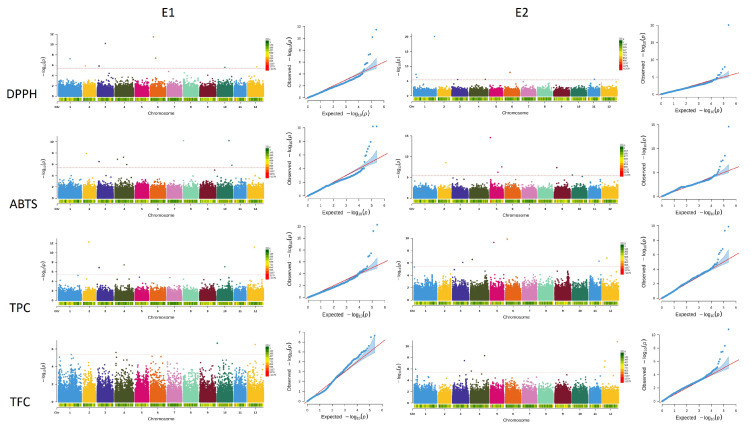
Manhattan plots represent a genome-wide association study regarding nutritional quality traits in 104 potato accessions across two growing seasons. E1 represents environment I and E2 represents environment II. Significant SNPs are plotted on the right for DPPH and ABTS activity along with total phenolic content and total flavonoid content. The *x*-axis represents each SNP’s chromosome number and position, while the *y*-axis represents the negative logarithm (−log10) of the *p*-values for each SNP in the Manhattan plot. The broken lines represent significant thresholds 4.02 × 10^−6^. The heat maps at the bottom of each chromosome represent SNP density. The QQ plot on the left shows the observed vs. expected −log10 values.

The SNPs and Indels utilized for GWAS spanned from 12,566 to 34,185 across the 12 chromosomes of potato genome, ensuring substantial genome coverage for association map-ping (Figure 1B). The Manhattan plots help to visualize GWAS results of color parameters (L*, a*, b*, C*, and H°) across the panel of diverse potato accessions in the two environments (Figure 4). The GWAS identified 36 SNPs that were significantly associated with the color parameters for fresh potatoes and potato powder in environment I, and 42 SNPs in environment II (Supplementary Table S1). In fresh potatoes, 2 SNPs were significantly associated with lightness (L*), 3 SNPs were linked to redness (a*), 6 SNPs with yellowness (b*), and 9 were recorded for hue angle (H°) in environment I. In comparison, in environment II, 6 SNPs were significantly linked with L*, 3 with a*, 6 with b*, and 9 significantly associated SNPs were detected for H°. In contrast, no significant SNP was detected for chroma C* in both environments (Figure 4A). Similarly, for potato powder, 6 significant SNPs were detected for lightness (L*), 3 for redness (a*), 6 for yellowness (b*), 9 for hue angle (H°), and no significant SNP was detected for chroma (C*) in potato powder in environment I, while in environment II, 7 SNPs were found as significantly linked with L*, 7 were detected for a*, 8 were detected for b*, 3 for C*, and 7 significantly associated with H° (Figure 4B). Thus, most SNPs detected were significantly associated with potato powder compared to fresh potato traits, suggesting a distinct pattern. These SNP results, especially non-synonymous SNPs on chromosome 5 associated with L*, play a crucial role in the flavonoid biosynthetic pathway. The G-A substitution leads to a change from alanine to valine amino acid at Chr5_ 46891767, which results in altering the function of the protein. Moreover, our results suggest that SNPs responsible for color diversity are present in almost all the chromosomes. Similarly, 50 SNPs were significantly linked with key phenotypic traits, including total phenolic content, total flavonoid content, and radical scavenging properties carried out by DPPH and ABTS assays (Supplementary Table S2). The GWAS analysis further revealed that 15 SNPs were significantly associated with DPPH activity, with 8 SNPs identified on chromosomes 1, 2, 3, 6, 10, and 12 in environment I, and 7 SNPs on chromosomes 1, 3, 4, 6, and 11 in environment II (Figure 5). For ABTS antioxidant capacity, 14 SNPs were elucidated, with 8 SNPs concerned with chromosomes 2, 3, 4, 8, and 10 in environment I and 6 SNPs located on chromosomes 2, 5, 9, and 10 in environment II. Similarly, 11 SNPs were identified for total phenolic content with 5 SNPs on chromosomes 2, 3, 4, 10, and 12 in environment I and 6 SNPs on chromosomes 3, 4, 5, 6, 11, and 12 in environment II. A total of 10 SNPs were reported for total flavonoid content, with 3 SNPs located on chromosomes 4, 10, and 12 in environment I, while 7 SNPs were on chromosomes 1, 3, 4, and 12 in environment II. The detected SNPs, especially the major SNPs identified on chromosomes 1, 3, 10, and 12 may further enhance the potential application of potato accessions with enhanced antioxidant and nutraceutical properties in the future.

### 2.9. Candidate Gene Analysis of Diverse Potato Population

#### 2.9.1. Candidate Genes for Color Parameters of Fresh Potato and Fine Powder

To detect candidate genes for color parameters in the potato genome, a database (https://plants.ensembl.org/Solanum_tuberosum/Info/Index, accessed on 20 December 2023) was used (Supplementary Table S1). The results show that three non-synonymous single-nucleotide variants (SNVs) were detected in the whole panel, with PGSC0003DMG400030097 being strongly linked with fresh potato color L*. This gene encodes the enzyme gamma-glutamyl transferase. It is a membrane-bounded enzyme that increases the availability of amino acids, especially cysteine for the intracellular production of glutathione. Glutathione is a key thiol antioxidant that plays a crucial role against oxidative stress and manages cardiovascular diseases, diabetes, and cancer [47,48]. The SNV on exon 1 of PGSC0003DMG400029504 was detected for potato powder color a*, and this gene encodes heat-resistant obscure (Hero) protein. This protein plays a vital role against pathogen attack, enhancing plant immune response [49]. A QTL for the potato color parameter chroma (C*) was detected on chromosome 3 [50]. A previous study revealed that the QTL for potato color was detected on chromosome 2 [51]; similarly, the present study identified non-synonymous SNV PGSC0003DMG400038862 for potato powder color C* on chromosome 2, encoding a Ring finger protein. This protein plays a crucial role in various infectious diseases such as inflammatory responses, cancers, and neurological disorders [52]. In potato, it has a role in drought tolerance [53]. Four synonymous SNVs were detected in the whole panel, of which PGSC0003DMG400012211 on exon 10 of chromosome 8 associated with potato powder color H°. It encodes the RB-binding protein, a tumor suppressor, which plays an important role in cell cycle regulation and tumor growth reduction [54]. Candidate gene PGSC0003DMG400020431 was detected for potato powder color a*, annotated as a conserved gene with an unknown function, PGSC0003DMG400042828 was significantly associated with fresh potato color L*, and PGSC0003DMG400037418 on chromosome 12 was associated with potato powder color b*, with an unknown function. Such genes with unknown functions are crucial for further research to know their exact role in potato biology.

A pleiotropic gene PGSC0003DMG400025098 present on chromosome 5 was significantly associated with potato powder color a* and H°, encoding the enzyme All-trans-retinol 13,14-reductase. It is essential for vitamin A metabolism and is involved in maintaining normal visual processes, particularly rhodopsin generation in the retina [55,56]. However, a candidate gene PGSC0003DMG400018939 on chromosome 10 was consistently detected across both environments as being strongly associated with the fresh potato color parameter b, indicating a significant impact on the yellow color in potatoes. It encodes an enzyme protein kinase, which plays a crucial role in cellular homeostasis, cancer, and immune responses [57,58]. Therefore, these genes need to be further studied to bring better changes in potato nutritional qualities.

#### 2.9.2. Candidate Genes for DPPH, ABTS, Total Phenolic, and Total Flavonoid Content

To detect candidate genes for total phenolic, total flavonoids, DPPH, and ABTS, in the potato genome, a database (https://plants.ensembl.org/Solanum_tuberosum/Info/Index, accessed on 20 December 2023) was used (Supplementary Table S2). The results identified a unique non-synonymous SNV at exon 1 of PGSC0003DMG400038170 on chromosome 10, significantly associated with ABTS in the potato genome. This SNV encodes a protein necessary to produce xanthoxin (Xan). Xanthoxin is involved in abscisic acid (ABA) biosynthesis, which might have a role in maintaining ABA levels, affecting plant growth and responses to various environmental stresses and other physiological functions [59]. Two pleiotropic genes were detected across the panel, including PGSC0003DMG400046306, detected on chromosome 10, which was significantly associated with DPPH and total phenolic contents. This gene encodes Gag-pol polyprotein that helps in human immune deficiency virus (HIV) replication and infects the host cell. Treating HIV involves inhibiting enzymes encoded by the pol protein such as reverse transcriptase, protease, and integrase [60]. But the other pleiotropic gene PGSC0003DMG400017104, detected on chromosome 3, was significantly associated with DPPH and ABTS with an unknown function, while a candidate gene PGSC0003DMG401026765, detected on chromosome 4, was significantly associated with total flavonoid content in both the tested environments. It is a conserved gene of unknown function, indicating that the same genes exist in many other species. This gene provides strong evidence for evaluating total flavonoid levels in potatoes. Such candidate genes, linked with various quality traits, must be investigated further to know their specific function. Therefore, experimental validation and functional characterization will be better for understanding the exact role of such candidate genes in potato physiology and quality improvement.

## 3. Discussion

### 3.1. Genetic Diversity of Total Phenolic, Total Flavonoid, and Antioxidant Properties of Potatoes

Potatoes possess distinct genetic backgrounds because of their genetic association and differentiation [7]. The current results demonstrated genetic variability influencing the nutritional composition, including fresh potato and powder color, total phenolic content, total flavonoid content, DPPH, and ABTS radical scavenging activities of 104 potato accessions grown across two environments. Based on the population structure analysis, the panel can be divided into two subpopulations. Across the two subpopulations, one comprises eleven accessions of uniform genotypic profiles. In contrast, the other subpopulation exhibits potato accessions with unique allelic profiles that represent shared allelic states, which indicates the existence of a wide genetic diversity of nutritional qualities across the two environments. Previous research identified total phenolic content in purple potatoes ranging from 162.19 to 510.20 mg GAE/100 g DW, and 113.37 to 114.63 in yellow-fleshed varieties [12]. These results revealed a consistency with our current findings, but our results showed slightly higher levels of total phenolic content in the colored and non-colored potato accessions (Table 2). The flavonoid is a diverse group of polyphenols responsible for various colors in potatoes and other fruits and vegetables [61]. They also exhibited a wide diversity, ranging from 6.0 to 229 mg CE/100g [62], similar to our current results, ranging from 19.22 to 160.32 mg CE/100g. It is well known that total phenolic and flavonoid contents in potatoes have strong DPPH and ABTS radical scavenging antioxidant potential [14,63]. Most of the reported data have similar ranges of both the DPPH and ABTS radical scavenging power. The levels of DPPH radical scavenging properties were detected as 8.67 µmol TE/g in red potatoes and 9.91 µmol/g in purple potatoes [64], whereas some lower levels of DPPH radical scavenging activities, such as 0.69 µmol TE/g in white and 2.78 µmol TE/g in colored potato accessions, were detected previously [45,65]. In comparison, our current results of white and colored potato accessions showed some higher levels of DPPH properties ranging from 1.95 to 29.30 µmol TE/g. However, the higher levels of antioxidant potential in our current investigations might be due to the use of a widespread diversity of genotypes evaluated in two diverse environmental conditions.

### 3.2. Diversity of Color in Potatoes

The current study examined yellow, red, and purple-fleshed potato accessions. The colored potato accessions demonstrated higher levels of total phenolic contents, total flavonoid contents, and antioxidant properties (DPPH and ABTS) in both tested environments. Among the colored potatoes, purple potatoes had higher total phenolic content, total flavonoid content, and antioxidant properties (DPPH and ABTS), across both environments, which is in agreement with previously reported data [45,46]. Therefore, it can be confidently stated that the potential of potato color stands as one the most effective ways to evaluate their nutritional qualities. Additionally, our phenotypic observations revealed that most white-fleshed potato accessions had a very dark red or purple peel. Such potato accessions might have extremely high bioactive components in peels compared with the flesh. It is well known that the peeling process decreases 40–60% of phenolic acids in yellow and about 80% in purple-fleshed varieties [5,66]. That is why the cooking of potatoes cannot ignore having peels on their surfaces, because the process of leaching will be more helpful in transferring the health-promoting bioactive compounds such as phenolic acids, flavonoids, and other nutrients from the peel to the flesh [67]. Our results also indicated that phenolic compounds had a positive correlation with the antioxidant properties of potatoes. So, this process will not only help to increase the quantity of health-beneficial components inside the potato flesh after cooking but could also play a vital role in the enhancement of various biological properties of potato flesh. Therefore, it is better to select potato varieties based on higher bioactive compounds and darker colors; such traits can bring a massive change in the nutritional qualities of potatoes.

### 3.3. Effect of Environments on Total Phenolic, Total Flavonoid, and Antioxidant Properties of Potatoes

Proper environmental conditions are better for maximum health-beneficial compounds and properties in potatoes. Previous research has shown that diverse environmental conditions have a considerable impact on total phenolic content and antioxidant properties in potatoes [33,68]. This is supported by the current results, which showed that total phenolic content, total flavonoid content, and antioxidant capacity in all the tested potato accessions were significantly affected by environmental conditions. Results revealed that environment II exhibited higher levels of all the tested nutritional quality traits than environment I. Specifically, DPPH radical scavenging activity increased by 37%, ABTS by 30%, and total phenolic content by 25%. The level of flavonoids in potatoes varies based on the skin and flesh color, genotype, and environmental conditions [9]. The present research showed that colored potatoes had higher flavonoid content than white potatoes, but the growing environments also had an effect, as its level in environment II was 5% higher than in environment I. The correlation analysis also revealed a strong positive correlation among the total phenolic content, total flavonoid content, and antioxidant properties (DPPH, and ABTS) within each environment and between the two tested environments, indicating phenolic compounds as the dominant antioxidant components. Based on the current results, it is well known that besides the diversity of color, environmental conditions can also significantly transform the phenolic and flavonoid contents, including their antioxidant potential in potatoes.

### 3.4. Genetic Basis of Potato Color, Total Phenolics, Total Flavonoids, and Antioxidant Properties

For the validation of the current findings and to know the exact marker responsible for transferring these quality traits in potatoes, single-nucleotide polymorphism (SNP) markers were generated using genome-wide association studies for potato accessions. The results demonstrated that 176 SNPs were significantly associated with color parameters, total phenolic content, total flavonoid content, DPPH, and ABTS radical scavenging properties. Various data from the literature concerning SNPs for potato flesh color present on chromosomes 1, 2, 3, 4, and 9 [50,69,70]. The current findings identified that most of the chromosomes, i.e., 1, 3, 10, and 12, may be beneficial to target for marker-assisted breeding to produce potato varieties with enhanced nutritional qualities and antioxidant properties.

The current study investigated a variety of candidate genes, of which several genomic regions were detected as pleiotropic across the genome. The candidate gene PGSC0003DMG400025316 encoding ATP-binding protein, detected on chromosome 4, was significantly associated with potato flesh color L* and the total flavonoid content. These proteins utilize the energy created by ATP hydrolysis and are thus involved in many cellular functions such as muscle contraction, active transport across membranes, protein synthesis, and signal transduction [71,72]. A candidate gene PGSC0003DMG400025100 was detected on chromosome 5, significantly associated with potato powder color b* and ABTS radical scavenging properties. This gene encodes the F-box family protein, which is essential for a variety of physiological activities such as transcriptional regulation, cell cycle transition, and signal transduction [73]. A study revealed that potato F-box family proteins are involved in tuber development as detected on chromosome 9 [74]. The candidate gene PGSC0003DMG400011551 detected on chromosome 12 was associated with potato powder color a* and total flavonoid content. This gene encodes a protein cyclin A, which is crucial in regulating the cell cycle and in controlling cancer [75]. The candidate gene PGSC0003DMG400042357, found on chromosome 12, is significantly associated with potato powder color a* and total phenolic content, while its function is unknown. Such candidate genes are considered as strongly associated SNPs for further characterization. However, it is further recommended to analyze these major SNP regions and map them to identify their exact role in potato biology.

Various functional proteins have previously been studied and associated with many quality traits in potatoes. The current study revealed that the candidate gene PGSC0003DMG400038784, detected on chromosome 1, was associated with DPPH radical scavenging properties. This gene encodes the late blight resistance protein, which plays a crucial role in preventing late blight fungal infection in potatoes caused by *phytophthora infestans* [76,77,78]. The candidate gene PGSC0003DMG401000586, detected on chromosome 3 and associated with total flavonoid content, encodes a subtilase family protein. Gene annotation, expression, and subcellular localization reveal a diverse function of potato subtilases, including pathogen defense. They also play a role against *Phytophthora infestans* infections in potatoes [79]. Another candidate gene present on chromosome 3, significantly associated with total phenolic content, encodes a basic leucine zipper (BZIP) domain class transcription factor. It is a member of the transcription regulator family involved in a variety of biological activities, including tissue development and stress response. It is also involved in stress responses in potatoes [80]. A candidate gene PGSC0003DMG400022867 detected on chromosome 11 related to fresh potato color b* encodes MADs-box protein. Various MADs-box proteins have been detected in potatoes, playing an important role in transcription regulation and promoting auxiliary meristem development [81,82]. A candidate gene PGSC0003DMG400030969 on chromosome 1 is significantly associated with potato powder L* encoding transcription factor. Various transcription factors have been identified in potatoes playing a crucial role in controlling biotic stress responses [83]. A candidate gene PGSC0003DMG400029867, detected on chromosome 3, was significantly associated with potato powder color b*, encoding amino acid transporter. This protein is also well known in potatoes and plays a role in various physiological processes in plants [84]. The gene PGSC0003DMG402027402, detected on chromosome 11, was significantly associated with potato powder color H° and encodes the disease resistance protein R3a, which generates resistance against the potato late blight pathogen known as *Phytophthora infestans* [85]. Interestingly, the current study revealed a candidate gene PGSC0003DMG400013752, detected on chromosome 12 and associated with total flavonoid content. It encodes the MYB domain class transcription factor. MYB transcription factor is the largest plant gene family that controls growth development, stress response, and anthocyanin synthesis. Many MYB genes reported in many tissues of potatoes create colorful phenotypes and regulate anthocyanin biosynthesis [86]. This gene indicated strong evidence and might be crucial in evaluating color and flavonoid content in potatoes. Overall, this study provides valuable genomic resources and targets for further advances in enhanced levels of phenolic content, flavonoid content, and antioxidant capacity under different environmental conditions, as well as in the selection of dark colors in the breeding field and marker-assisted selection for potato quality improvement.

## 4. Materials and Methods

### 4.1. Plant Materials and Preparation of Whole-Meal Potato Powder

A diverse panel of 104 tetraploid potato accessions was cultivated in Huajiachi Campus at Zhejiang University in Hangzhou, China, in 2019 (environment I) and 2020 (environment II), employing a randomized complete block design with two replications per accession. The soil type is fluvio-marine yellow loamy soil, which contained around 150 mg kg^−1^ alkaline-hydrolysable N, 30 mg kg^−1^ available P, and 180 mg kg^−1^ available K. The weather data, i.e., rainfall, sunshine, and minimum, maximum, and average temperatures, from March to June during potato growth across two years are shown in Supplementary Figures S1 and S2. Fertilizer and field management were followed with the conventional practice of potatoes. Ten plants were cultivated per replicate for each accession. The young and fresh leaves were collected for DNA extraction in fresh Eppendorf tubes for each accession. After harvest in mid-June, 15 potato tubers were chosen randomly from each accession and washed cleanly. About 300 g of the cleaned potato tubers were peeled and cut into small slices. The potato pieces were rapidly treated with liquid nitrogen and then transferred to a freeze dryer (Boyikang Experimental Instrument, Beijing, China) at −50 °C, undergoing a 72 h freeze-drying process. The resultant freeze-dried potato pieces were then ground into a fine powder, carefully placed into sealed plastic bags, and stored at 4 °C for further analysis.

### 4.2. Methanolic Extraction

Samples were extracted using the protocols described by Ru et al. and Xu et al. [45,87], with minor modifications. Initially, 0.2 g of potato powder was mixed with 15 mL of 80% methanol. To ensure maximum extraction efficacy, the samples underwent two thorough mixing rounds using a premium mechanical shaker (HZ-9210K Desktop frozen oscillator, Suzhou, China) for at least 1 h each time. Next, the mixture was centrifuged for 10 min at 3000 rpm to separate the supernatant from the solid particles. We then collected the supernatant of each sample and subjected it to a second round of centrifugation at 3000 rpm for 5 min to remove any remaining particles. Finally, the purified supernatants were transferred into fresh tubes and stored at 4 °C for further analysis. 

### 4.3. Determination of Color Parameter of Potato Fine Powder

The color parameters for potato flesh and fine powder were measured by a spectrophotometer, NS800 (Shenzhen 3NH Technology Co., Ltd., Shenzhen, China). The color parameters were expressed as CIELAB parameters L*, a*, and b*, where L* indicates lightness or darkness. A higher positive L* indicates the lightness while higher negative L* indicates the darkness of the sample. The a* indicates the difference between redness and greenness. The highest positive a* indicates red while the higher negative a* indicates the green color of the sample. The higher positive b* indicates yellow while the higher negative b* indicates a bluer color. Furthermore, the C* (chroma) represents the color intensity or saturation, calculated as C* = (a*^2^ + b*^2^)^1/2^, while the H° (hue angle) indicates the sample color, which is calculated as H° = tan − 1(b*/a*), where 0° or 360° represents red-purple, 90° represents yellow, 180° represents green, and 270° represents the blue color [88,89].

### 4.4. Determination of Total Phenolic Content (TPC)

To evaluate the total phenolic compounds in potato powder samples, a Folin–Ciocalteu assay was performed, as described by Ainsworth and Gillespie [90], with some modifications. A freshly prepared solution of 10-fold diluted Folin–Ciocalteu reagent (1.5 mL) was mixed with appropriately diluted samples or standard solutions (200 µL) and allowed to react for 5 min. Next, the mixture was neutralized with a saturated sodium carbonate solution (75 g/L, 1.5 mL). After incubating the reaction mixture in darkness at room temperature for 2 h, we measured its absorbance at 725 nm using a spectrophotometer in duplicate for each sample across the two environments. To ensure the accuracy of our results, we performed each measurement in duplicate. The phenolic contents were quantified in terms of milligrams of gallic acid equivalent per 100 g (mg GAE/100 g) of the dry weight of potato powder, as gallic acid was used as a standard.

### 4.5. Determination of Total Flavonoid Content (TFC)

To determine the flavonoid content, the aluminum chloride method described by Xu et al. [87] was employed. First, three solutions were prepared; solution-1 containing 5% NaNO_2_, solution-2 containing 10% AlCl_3_.6H_2_O, and 1 M NaOH. Next, 150 µL of solution-1 was mixed with 1 mL of properly diluted samples or standard solutions and allowed to react for 5 min. Subsequently, 150 µL of solution-2 was added to the above mixture and kept for a 5 min reaction. After the reaction, 1 mL of 1 M NaOH solution was mixed, followed by 3 mL of ddH_2_O. Finally, the mixture was kept in darkness at room temperature for 45 min before measuring the absorbance of yellow color at 510 nm using a spectrophotometer. The total flavonoid contents of all the tested samples were analyzed in duplicate and expressed as milligrams of catechin acid equivalent per 100 g (mg CE/100 g) of potato powder with catechin acid used as a standard for flavonoid content.

### 4.6. Determination of DPPH Radical Cation Scavenging Activity

The DPPH (2,2-Diphenyl-1-picrylhydrazyl) test was used to examine the antioxidant properties of potato samples as described by Brand-Williams et al. [91]. Firstly, 3 mL of 100 µM DPPH solution was mixed with 200 µL of either the sample or a standard solution. The reaction mixture was then kept in a dark room at room temperature for 45 min. Then, the absorbance value at 517 nm was measured using a spectrophotometer in duplicate for all the tested samples. The DPPH scavenging activity for both the samples and the standard (Trolox) was calculated using the formula: DPPH (%) = (1 − Asample/A control) × 100. The results were expressed as micromoles of Trolox equivalent of DPPH radical scavenging activity per gram (µmol TE/g) of potato powder.

### 4.7. Determination of ABTS Radical Scavenging Activity

The ABTS (2,2-azino-bis-(3-ethylbenzothiazoline-6-sulphonic acid) radical cation scavenging activity was measured following the procedures of Re et al. and Xu et al. [87,92]. The ABTS working solution (ABTS•+) was prepared by mixing 7 mM ABTS and 2.45 mM potassium persulfate (*v*/*v*, 2:1) and stirring it at room temperature for 12–16 h before use. The absorbance of the working solution was adjusted to 0.700 ± 0.020 at 734 nm by adding distilled water. Next, we mixed 3.9 mL of the ABTS•+ working solution with 100 µL of the sample or standard solution and kept it in the dark for 45 min at room temperature. The absorbance was measured at 734 nm in duplicates for each sample using a spectrophotometer. Finally, we expressed the results as micromoles of Trolox equivalent of ABTS radical scavenging activity per gram (µmol TE/g) of potato powder as Trolox was used as a standard.

### 4.8. Genomic DNA Extraction and Resequencing

After the emergence of 104 germplasms, the healthy and non-degraded plants were selected to take fresh leaves. Genomic DNA was extracted from 100 mg of fresh young potato leaves employing the DNeasy Plant Pro Kit (Qiagen, Valencia, CA, USA), and then double-digest Restriction-site Associated DNA sequencing (ddRAD-seq) was conducted using the restriction enzymes SacI and MseI. Sequencing of 300–500 bp paired-end reads of the library was conducted by Genergy Bio Corporation (Shanghai, China) using HiSeq PE150 (Illumina, San Diego, CA, USA).

### 4.9. SNP Calling, Filtering, and Markers Classification

The raw Illumina readings were demultiplexed with the process_radtags program in Stacks v2.029 [93]. FASTQ was used to construct the quality distribution plots, and the filtered data were aligned with the reference potato genome (*S. tuberosum* v4.03) using BWA-MEM as the default setting [94]. SAMtools (version 1.7) was used to sort BAM files generated by BWA output and delete duplicates to maintain reads with a mapping quality greater than Q10 [95]. Genome Analysis Toolkit (GATK, V3.4-46) was then used to call SNPs and Indels based on the sorted and filtered alignment data [96]. SNPs and Indels considered ‘PASS’ by GATK, with a missing rate of less than 0.15 and a minor allele frequency exceeding 0.05, were kept for further investigation.

### 4.10. Population Structure Analysis

The neighbor-joining tree, principal component analysis, and structure plots were used to infer the population structure of 104 potato accessions. The neighbor-joining tree was constructed using Molecular Evolutionary Genetics Analysis (MEGA) software (version 11) based on a pairwise distance matrix derived from the simple matching distance for 226,487 SNPs. Principal component analysis was performed using GCTA software (version 1.94) [97]. The first two principal components were used for PCA plots. The ADMIXTURE (version 1.23) [98] was used to estimate the optimum number of clusters for the 104 potato accessions. ADMIXTURE includes a cross-validation procedure that allows the user to identify the value of K for which the model has the best predictive accuracy, as determined by holding out data points. A good value of K will exhibit a low cross-validation error compared to other K values. The LD (r^2^) between pairs of markers was calculated using PopLDdecay software (version 3.42) [99]. When r^2^ declined to half of its maximum value, the distance across the chromosome was determined as the distance of LD decay [100].

### 4.11. Genomic-Wide Association Study (GWAS)

After reducing the heterozygosity rate (<0.2), the remaining 226,487 SNPs and 22,115 Indels were merged. The LD KNNi was used to identify the missing genotypes in the Tassel. The primary principle component was included as the fixed covariate, and the genetic kinship as the random effect in the fixed-and-random model-circulating probability unification (FarmCPU) model in the rMVP software (version 1.1.0)for association analysis [101]. The genome-wide significant threshold of the GWAS (*p*-value = 4.02 × 10^−6^) was determined by 1/n (n being the number of markers). Significant markers were annotated using ANNOVAR based on the PGSC_DM_V403_genes.gff file (S.tuberosum genome v.4.0.3). The Manhattan, QQ, and SNP-density plots for the GWAS were created using the R package CMplot (version 3.3.1).

### 4.12. Statistical Analysis

Statistical analysis, including descriptive statistics (average, minimum, and maximum) and Pearson correlation, for this study was conducted using IBM SPSS Statistics 27. Analysis of variance (ANOVA) was conducted in a generalized linear model (GLM) using SAS (SAS Institute, Cary, NC, USA, version 9.4). Distribution analysis and normality tests based on the Shapiro–Wilk test [102] were conducted using JMP Pro 17 statistical software. Box plot analysis was performed using the ggplot2 package (version 2.2.0) in R studio.

## 5. Conclusions

Potato breeding requires a diversity of nutritional traits to meet market demands. This research assessed the genetic diversity of various potato genotypes based on nutritional quality traits, including color parameters, total phenolic content, total flavonoid content, DPPH, and ABTS radical scavenging potential in two environments. Population structure revealed two subpopulations comprising a wide variety of genotypes. Environment II has more bioactive compounds and antioxidant potential than environment I. All the traits studied showed that colored potato accessions are superior to white potato accessions. The GWAS identified significant SNPs and candidate genes; specifically, those present on chromosome 1 for DPPH activities and chromosome 3 for total flavonoids have the potential for resistance breeding. The pleiotropic genes on chromosomes 3, 5, and 10 provided stronger evidence of the genetic basis of potato color, total phenolic content, total flavonoid content, and antioxidant properties. Therefore, the study recommends novel opportunities to enhance potato bioactive compounds and properties via strategic breeding and marker-assisted selection for superior varieties and the most suitable alleles for potato quality improvement.

## Figures and Tables

**Table 1:** Types of genomic variants observed in potato populations.

SNPs	Number	Indels	Number
Total SNPs	1,101,368	Total Indels	141,656
SNPs in exon	75,533	Indels in exon	3359
SNPs in intron	128,133	Indels in intron	21,696
SNPs in UTR5	15,510	Indels in UTR5	3420
SNPs in UTR3	22,621	Indels in UTR3	5283
SNPs in intergenic region	682,604	Indels in intergenic region	76,580
Upstream	84,244	Upstream	14,473
Downstream	75,480	Downstream	13,155
Non-synonymous SNPs	39,547	Splicing Indels	92
Synonymous SNPs	34,814	Stop-gain Indels	80
Splicing SNPs	216	Stop-loss Indels	9
Stop-gain SNPs	1064	Frameshift Indels	1550
Stop-loss SNPs	125	Nonframeshift Indels	1721

**Table 2:** Descriptive statistics, analysis of variance (ANOVA), and broad-sense heritability of color parameters of 104 potato accessions in respect to fresh potatoes and potato fine powder.

		Color Parameters (Fresh Potato)					Color Parameters (Potato Fine Powder)				
		L*	a*	b*	C*	H°	L*	a*	b*	C*	H°
Environment I											
Colored potato	Mean ± SD	45.8 ± 15.2	13.6 ± 1.31	1.74 ± 7.89	15.5 ± 1.16	128.5 ± 155.9	69.9 ± 7.52	10.4 ± 0.62	1.26 ± 6.31	11.7 ± 0.63	125.8 ± 175.6
	C.V.	33.1	9.70	454.6	7.51	121.4	10.8	5.99	499.7	5.45	139.5
	Range	25.4–57.8	11.9–15.1	−16.84	14.2–17.4	20.0–329.7	61.3–75.4	9.79–11.0	−11.57	11.1–12.4	22.1–328.6
Non-colored potato	Mean ± SD	68.6 ± 3.10	0.23 ± 1.32	20.8 ± 5.27	20.3 ± 5.28	89.3 ± 2.03	89.5 ± 1.79	1.05 ± 0.43	12.8 ± 1.79	12.9 ± 1.79	85.3 ± 1.94
	C.V.	4.51	562.8	25.4	25.3	2.27	2.01	41.7	14.0	14.0	2.27
	Range	60.6–75.3	−19.85	11.0–30.6	11.0–31.0	68.7–91.9	82.5–92.8	0.10–2.67	7.03–16.5	7.24–16.5	76.1–89.4
Total	Mean ± SD	67.9 ± 5.60	0.66 ± 2.68	20.2 ± 6.30	20.8 ± 5.29	90.5 ± 26.4	88.9 ± 3.97	1.34 ± 1.67	12.4 ± 2.81	12.8 ± 1.78	86.6 ± 26.2
	C.V.	5.60	409.0	31.2	25.5	29.1	4.47	125.2	22.6	13.9	30.3
	Range	25.4–75.3	−30.7	−39.35	11.0–31.0	20.0–329.7	61.3–92.8	0.10–11.0	−22.48	7.24–16.5	22.1–328.6
Environment II											
Colored potato	Mean ± SD	50.6 ± 1.82	15.7 ± 1.07	5.82 ± 0.90	16.8 ± 0.90	20.5 ± 2.86	69.5 ± 2.14	13.1 ± 1.79	1.58 ± 2.83	13.3 ± 1.42	187.6 ± 241.4
	C.V.	3.61	6.88	15.5	7.11	14.0	3.09	13.7	178.9	10.7	128.7
	Range	48.8–52.9	14.4–16.6	4.96–6.97	15.6–18.2	16.7–22.9	68.0–71.1	11.8–14.3	−4.01	12.3–14.4	16.9–358.3
Non-colored potato	Mean ± SD	66.6 ± 2.75	0.28 ± 0.49	20.5 ± 5.11	20.5 ± 5.12	89.4 ± 1.36	87.4 ± 4.08	1.18 ± 0.77	12.0 ± 1.42	12.0 ± 1.49	84.5 ± 2.79
	C.V.	57.2	174.8	25.0	25.0	1.52	4.67	65.3	11.9	12.4	3.30
	Range	57.2–74.3	−2.85	9.42–32.0	9.43–32.1	85.3–93.4	68.8–92.0	0.12–5.51	7.95–17.9	7.98–18.7	72.9–89.5
Total	Mean ± SD	66.3 ± 3.54	0.59 ± 2.22	20.2 ± 5.46	20.4 ± 5.09	88.0 ± 9.82	87.0 ± 4.81	1.44 ± 1.90	11.7 ± 2.09	12.1 ± 1.49	86.7 ± 29.5
	C.V.	5.35	377.0	27.1	25.0	11.2	5.53	132.0	17.8	12.4	34.0
	Range	48.8–74.3	−0.73–16.6	4.96–32.0	9.43–32.1	16.7–93.4	68.0–92.0	0.12–14.3	−18.32	7.98–18.7	16.9–358.3
Anova											
Source	df	Mean Square									
Genotype	93	38.3 **	17.5 **	106.5 **	93.0 **	4.17 **	46.4 **	11.0 **	14.3 **	6.87 **	14.3 **
Year	1	395.3 **	0.02	7.54	11.8	0.002	400.7 **	1.44 **	80.4 **	62.6 **	38.7 **
G × E	93	7.94 **	0.56 **	10.9 **	11.0 **	2.16 **	16.9 **	0.57 **	2.84 **	2.97 **	6.34 **
Error		2.98	0.07	2.57	1.84	0.51	0.02	0.00	0.01	0.02	0.03
H^2^		0.09	0.96	0.84	0.79	0.61	0.10	0.85	0.15	0.09	0.24

Summary statistics represent mean of two repeats; L*, lightness; a*, redness; b*, yellowness; C*, chroma; H°, hue angle; mean square values represent ** *p* < 0.01; df, degree of freedom; G × E, genotype–environment interaction; and H^2^, broad-sense heritability.

**Table 3:** Summary statistics of total phenolic content, total flavonoid content, DPPH, and ABTS properties of diverse potatoes across two environments.

		DPPH ^a^	ABTS ^a^	TPC	TFC
Environment I					
Colored potato	Mean ± SD	19.8 ± 6.17	23.8 ± 3.88	395.3 ± 116.6	95.9 ± 80.6
	C.V. %	31.2	16.3	29.5	84.1
	Range	15.3–26.8	19.8–27.5	306.9–527.4	22.9–182.5
Non-colored potato	Mean ± SD	7.23 ± 2.76	10.2 ± 2.80	149.2 ± 58.9	33.4 ± 23.2
	C.V. %	38.2	27.5	39.5	69.4
	Range	1.95–17.7	4.23–20.3	36.9–340.5	19.2–160.3
Total	Mean ± SD	7.61 ± 3.59	10.6 ± 3.67	156.7 ± 73.9	35.3 ± 27.8
	C.V.	47.2	34.5	47.2	78.7
	Range	1.95–26.8	4.23–27.5	36.9–527.4	19.2–182.5
Environment II					
Colored potato	Mean ± SD	27.3 ± 2.86	33.1 ± 2.74	480.8 ± 72.1	168.5 ± 54.4
	C.V. %	10.5	8.29	15.0	32.3
	Range	25.3–29.3	31.2–35.0	429.9–531.8	130.0–206.9
Non-colored potato	Mean ± SD	10.0 ± 2.90	13.4 ± 2.80	189.8 ± 70.1	34.2 ± 18.5
	C.V. %	29.0	20.9	37.0	54.0
	Range	4.46–19.0	7.47–24.0	40.5–368.4	19.3–128.8
Total	Mean ± SD	10.4 ± 3.84	13.8 ± 4.01	196.1 ± 81.8	37.1 ± 27.5
	C.V. %	36.9	29.0	41.7	74.0
	Range	4.46–29.3	7.47–35.0	40.5–531.8	19.3–206.9
Anova					
Source	df	Mean Square			
Genotype	93	36.1 **	40.9 **	18,073.8 **	1945.0 **
Year	1	825.4 **	1003.9 **	186,100.5 **	1446.4 **
G × E	93	12.6 **	13.3 **	3156.7 **	688.7 **
Error		0.91	1.40	8.83	1.62
H^2^		0.04	0.04	0.09	0.48

^a^ DPPH, 2,2-Diphenyl-1-picrylhydrazyl; ABTS, 2,2-azino-bis-(3-ethylbenzothiazoline-6-sulphonic acid) expressed as µM TE/g of potato powder; TPC, total phenolic content expressed as mg GAE/100 g; TFC, total flavonoid content expressed as mg/g CE/100 g of potato powder; mean square values represent ** *p* < 0.01; df, degree of freedom; G × E, genotype–environment interaction; and H^2^, broad-sense heritability.

**Table 4:** Pearson correlation amongst L*, a*, b*, C*, H°, DPPH, ABTS, TPC, and TFC, of 104 potato samples in two environments.

		Potato Flesh					Potato Powder								
		L*	a*	b*	C*	H°	L*	a*	b*	C*	H°	DPPH	ABTS	TPC	TFC
Potato flesh	L*	0.65 **	−0.67 **	0.22 *	0.02	0.68 **	0.42 **	−0.64 **	0.37 **	−0.27 *	−0.30 **	−0.54 **	−0.57 **	−0.44 **	−0.46 **
	a*	−0.74 **	0.95 **	−0.27 **	0.02	−0.99 **	−0.52 **	0.88 **	−0.69 **	0.13	0.45 **	0.66 **	0.72 **	0.53 **	0.71 **
	b*	0.42 **	−0.43 **	0.82 **	0.96 **	0.33 **	0.31 **	−0.43 **	0.47 **	0.18	−0.19	−0.18	−0.36 **	−0.16	−0.24 *
	C*	0.06	−0.05	0.88 **	0.79 **	0.04	0.16	−0.17	0.27 **	0.24 *	−0.06	0.02	−0.16	−0.01	−0.03
	H°	−0.62 **	0.28 **	−0.36 **	−0.05	0.97 **	0.53 **	−0.90 **	0.71 **	−0.13	−0.50 **	−0.66 **	−0.73 **	−0.53 **	−0.72 **
Potato powder	L*	0.72 **	−0.83 **	0.52 **	0.16	−0.49 **	0.54 **	−0.78 **	0.12	−0.51 **	−0.25 *	−0.46 **	−0.56 **	−0.40 **	−0.46 **
	a*	−0.67 **	0.92 **	−0.54 **	−0.22 *	0.21 *	−0.91 **	0.90 **	−0.52 **	0.36 **	0.51 **	0.65 **	0.72 **	0.50 **	0.73 **
	b*	0.66 **	−0.73 **	0.61 **	0.30 **	−0.49 **	0.58 **	−0.61 **	0.62 **	0.56 **	−0.51 **	−0.45 **	−0.55 **	−0.33 **	−0.52 **
	C*	0.08	−0.15	0.31 **	0.31 **	−0.03	−0.08	−0.01	0.71 **	0.34 **	0.11	0.20	0.13	0.21 *	0.21 *
	H°	−0.63 **	0.30 **	−0.33 **	−0.01	0.99 **	−0.46 **	0.20 *	−0.50 **	−0.05	−0.53 **	0.40 **	0.42 **	0.35 **	0.53 **
	DPPH	−0.53 **	0.58 **	−0.46 **	−0.19	0.41 **	−0.73 **	0.66 **	−0.47 **	−0.01	0.37 **	0.50 **	0.72 **	0.56 **	0.69 **
	ABTS	−0.46 **	0.65 **	−0.46 **	−0.22 *	0.29 **	−0.61 **	0.64 **	−0.50 **	−0.07	0.28 **	0.60 **	0.52 **	0.61 **	0.76 **
	TPC	−0.47 **	0.57 **	−0.33 **	−0.05	0.38 **	−0.74 **	0.63 **	−0.34 **	0.15	0.35 **	0.74 **	0.57 **	0.73 **	0.54 **
	TFC	−0.43 **	0.42 **	−0.43 **	−0.19	0.55 **	−0.52 **	0.40 **	−0.36 **	0.02	0.47 **	0.71 **	0.53 **	0.61 **	0.48 **

* *p* < 0.05, ** *p* < 0.01; the lower diagonal represents the results concerning environment I, while the upper diagonal represents results from environment II; L*, lightness; a*, redness; b*, yellowness; C*, chroma; H°, hue angle; DPPH, 2,2-Diphenyl-1-picrylhydrazyl; ABTS, 2,2-azino-bis-(3-ethylbenzothiazoline-6-sulphonic acid); TPC, total phenolic content; TFC, total flavonoid content.

## Data Availability

The original contributions presented in this study are included in the article/Supplementary Material. Further inquiries can be directed to the corresponding author(s).

## Supplementary Materials

The following supporting information can be downloaded at: https://www.mdpi.com/article/10.3390/ijms252312795/s1.
