# Supplementary material for: Genetic Diversity and Genome-Wide Association Study of Total Phenolics, Flavonoids, and Antioxidant Properties in Potatoes (Solanum tuberosum L.)
Source: Int J Mol Sci. 2024 Nov 28;25(23):12795. doi: 10.3390/ijms252312795 (PMC11640895; doi:10.3390/ijms252312795)
Supplement: Supplementary file 1 [file ijms-25-12795-s001.zip › ijms-3312078-Supplementary Figures.pdf]

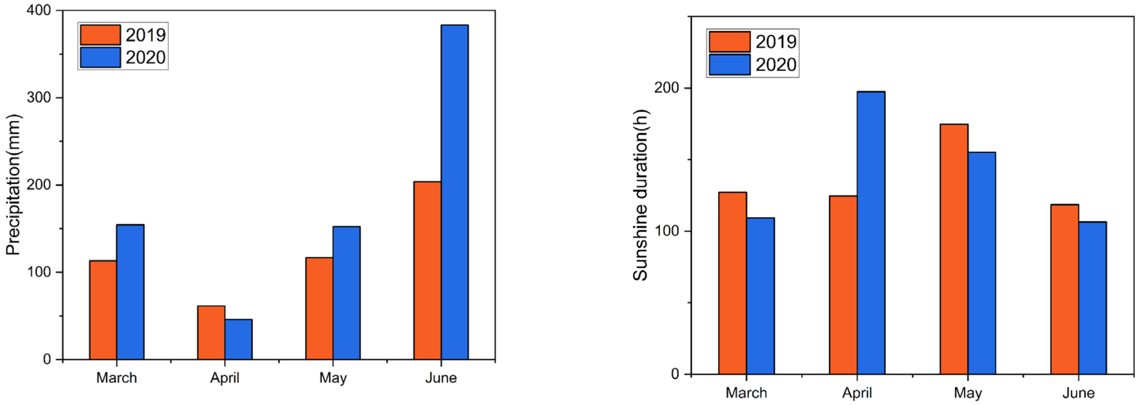

(A) **(B)**

Supplementary Figure S1. Monthly rainfall (A) and sunshine (B) from March to June during potato growth in two years.

(A)

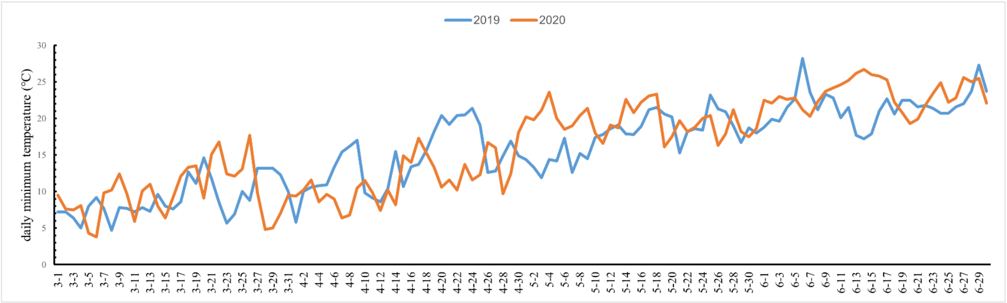

(B)

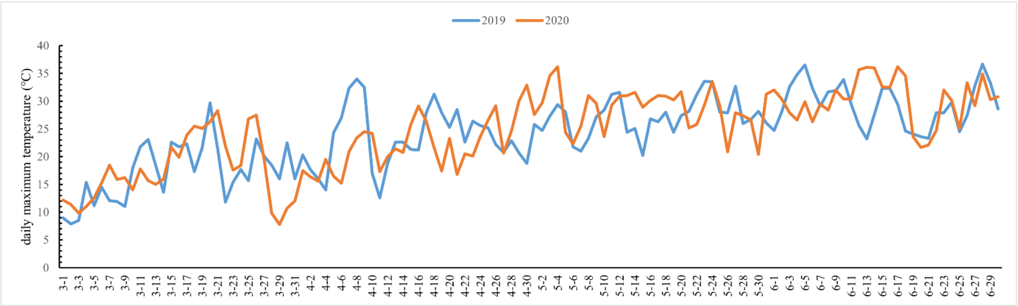

(C)

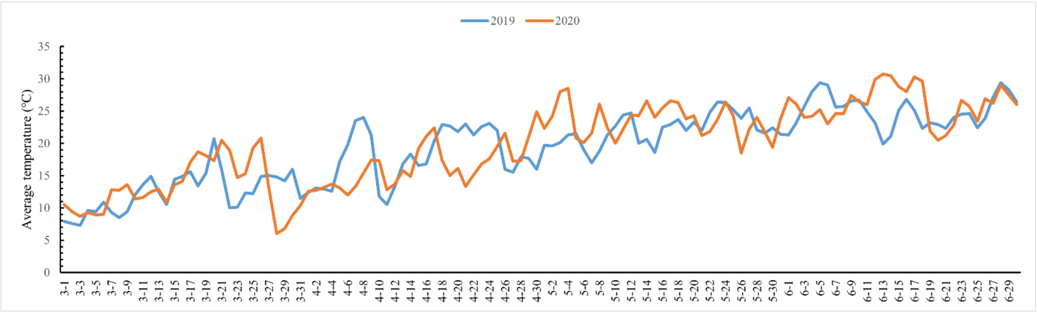

Supplementary Figure S2. Daily minimum (A), maximum (B) and average (C) temperatures from March to June during potato growth in two years
